# High-pressure balloon dilatation of primary obstructive megaureter in children: a systematic review

**DOI:** 10.1186/s12894-023-01199-5

**Published:** 2023-03-03

**Authors:** Liisi Ripatti, Hanna-Reeta Viljamaa, Anna Suihko, Niklas Pakkasjärvi

**Affiliations:** 1 Department of Pediatric Surgery, Turku University Hospital, Savitehtaankatu 5, PL 52, 20520 Turku, Finland; 2 Department of Pediatric Surgery, Uppsala Akademiska Sjukhuset, Uppsala, Sweden

**Keywords:** POM, Hydroureteronephrosis, Infant, Endoscopic, Treatment

## Abstract

**Objective:**

We aimed to evaluate the effectiveness and complication rates of endoscopic high-pressure balloon dilatation (HPBD) in treating primary obstructive megaureter (POM) in children based on current literature. Specifically, we wanted to clarify the evidence on the use of HPBD in children under one year of age.

**Methods:**

A systematic search of the literature was performed via several databases. The preferred reporting items for systematic reviews and meta-analyses guidelines were followed. The primary outcomes studied in this systematic review were the effectiveness of HBPD in relieving obstruction and reducing hydroureteronephrosis in children. The secondary outcome was to study the complication rate of endoscopic high-pressure balloon dilatation. Studies that reported one or both of these outcomes (n = 13) were considered eligible for inclusion in this review.

**Results:**

HPBD significantly decreased both ureteral diameter (15.8 mm [range 2–30] to 8.0 mm [0–30], *p* = 0.00009) and anteroposterior diameter of renal pelvis (16.7 mm [0–46] to 9.7 mm [0–36],* p* = 0.00107). The success rate was 71% after one HPBD and 79% after two HPBD. The median follow-up time was 3.6 years (interquartile range 2.2–6.4 years). A complication rate of 33% was observed, but no Clavien–Dindo grade IV–V complications were reported. Postoperative infections and VUR were detected in 12% and 7.8% of cases, respectively. For children under one year of age, outcomes of HPBD seem to be similar to those in older children.

**Conclusions:**

This study indicates that HPBD appears to be safe and can be used as the first-line treatment for symptomatic POM. Further comparative studies are needed addressing the effect of treatment in infants, and also long-term outcomes of the treatment. Due to the nature of POM, identifying those patients who will benefit from HPBD remains challenging.

## Introduction

Primary obstructive megaureter (POM) is a descriptive term for the dilated ureter secondary to distal segmental obstruction. It accounts for up to 10% of prenatal hydronephrosis detected [1, 2]. Age at diagnosis has decreased with the use of more accurate prenatal ultrasound techniques, with presentation moving from symptomatic to asymptomatic [3]. While most cases of POM may be managed conservatively, controversy exists regarding surgical intervention. Ureteral reimplantation in early infancy is not problem free and open intervention is commonly postponed, if possible, for optimal outcomes. In a cohort of children with a mean age of 4.9 years, the overall success rate for ureteral reimplantation surgery in primary megaureter was 82% [4]. While not all patients with POM diagnosed in infancy will get symptomatic, part of those who do may get so early. Paediatric urologists are often hesitant to operate on the immature bladder not to induce unwanted developmental disturbances. Still, symptomatic patients may require interventions to protect the developing kidneys, and occasionally one must balance the risks associated with kidney damage vs. bladder disturbance. The risk of complications associated with open ureteral reimplantation spawned efforts to find alternative interventions with lower complication risk. To that end, mini-invasive techniques have been developed.

Endoscopic high-pressure balloon dilatation (HPBD) was introduced in 1998 and has, through the development of the technique, become a viable alternative to the treatment of symptomatic POM [5]. High-quality comparative studies comparing endoscopic high-pressure dilatation to traditional open ureteral reimplantation in infants are scarce. Further, long-term benefits remain debatable within the paediatric urologic community. Doudt et al. [6] systematically summarised data on HPBD for treating POM and concluded that it is an alternative for patients over 1 year of age. Here, we aimed to systematically review the current data on high pressure balloon dilatation (endoscopic treatment) of POM, emphasising outcomes in a systematic review including a quantitative analysis of treatment outcomes. We specifically wanted to clarify the effect of HPBD on relieving obstruction and alleviating hydroureteronephrosis. Further, we wanted to determine adverse effects in the form of postoperative infections, vesicoureteral reflux (VUR), and overall complication rate. Additionally, we aimed to determine whether there is sufficient evidence to support the use of HPBD in treating children under one year of age.

## Methods

### Registration and search strategy

**Fig. 1:**
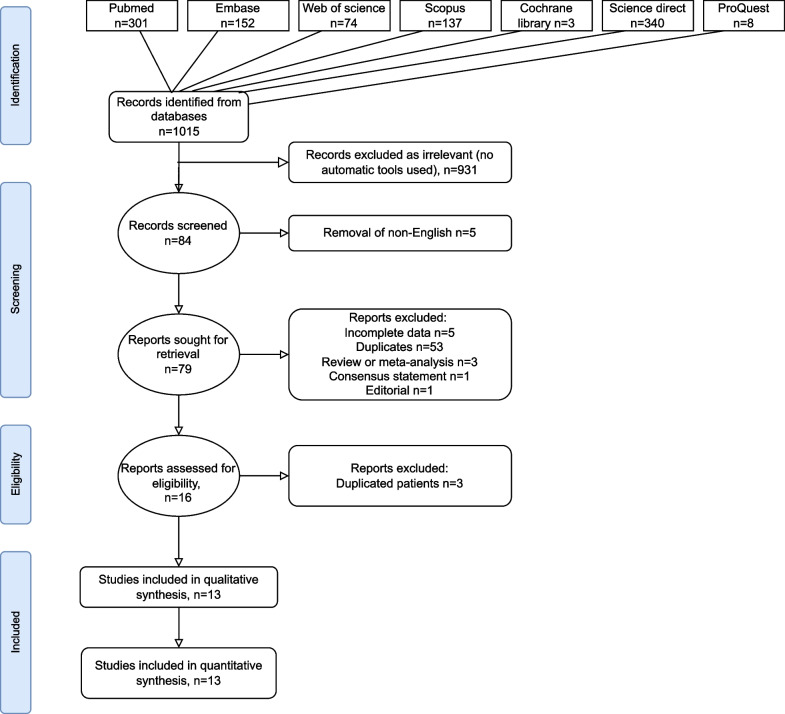
Selection of the relevant studies using the preferred reporting items for systematic review and meta-analysis (PRISMA) flow diagram

This systematic review was registered in the International prospective register of systematic reviews (PROSPERO) on May 22nd, 2022 (CRD42022334484). The Preferred Reporting Items for Systematic Reviews and Meta-Analyses (PRISMA) guidelines were followed [7]. The following databases were systematically searched by two of the investigators (LR and NP); PubMed, Embase, Scopus, ProQuest, Cochrane Library, Science Direct, and Web of science. The search terms used to identify the relevant studies were ("obstruct"[All Fields] OR "obstructed"[All Fields] OR "obstructing"[All Fields] OR "obstruction"[All Fields] OR "obstructions"[All Fields] OR "obstructive"[All Fields] OR "obstructs"[All Fields]) AND ("megaureter"[All Fields] OR "megaureters"[All Fields]) AND ("child"[MeSH Terms] OR "child"[All Fields] OR "children"[All Fields] OR "child s"[All Fields] OR "children s"[All Fields] OR "childrens"[All Fields] OR "childs"[All Fields]) AND ("therapeutics"[MeSH Terms] OR "therapeutics"[All Fields] OR "treatments"[All Fields] OR "therapy"[MeSH Subheading] OR "therapy"[All Fields] OR "treatment"[All Fields] OR "treatment s"[All Fields]). We included only studies concerning children. We also performed snowballing and reverse snowballing to identify articles that were not included in the abovementioned databases. We removed irrelevant studies, studies that were not published in English, those with incomplete data, duplicates, review articles and meta-analyses, consensus statements, editorials, and studies with duplicated patients (see Fig. 1). The remaining articles (n = 13) were analysed.

### The definition of PICOS used in this study

Participants: Children with symptomatic POM.

Intervention: HPBD of POM.

Comparators: None available.

Outcomes: Effect of treatment on ureterorenal parameters, and complications.

Study design: All studies on HPBD for treatment of symptomatic POM were included.

### Eligibility criteria

We included studies on patients < 18 years of age with the diagnosis of POM documented using both ultrasound and MAG3 renogram. We included only studies with HPBD as the intervention. The main outcomes studied in this systematic review were the effectiveness of endoscopic high pressure balloon dilatation in relieving obstruction and reducing hydroureteronephrosis. The secondary outcome was to study the complication rate of HPBD. Studies that reported one or both outcomes were judged as eligible for inclusion in this review.

### Synthesis of the data

The data was gathered independently by AS, LR, and HRV. Any ambiguity between these authors was resolved in discussions with the senior author NP. Data was systematically collected on the study outcomes, baseline information of the publication, the study design, the number of patients and of POM treated, preoperative and postoperative ultrasound, MAG3 renogram, and voiding cystourethrogram (VGUC) results, the number and type of complications, and the quality of the studies included.

### Quality assessment

All authors assessed the quality of the included studies by using preplanned questions specific from a urological standpoint (ureteral diameter pre- and postoperative, renal pelvis diameter pre-and postoperative, renogram differential function and half-times, infections, complications, post-operative VUR, reoperations). Further methodological quality assessment was performed using the Downs and Black scale [8], rated by two authors (LR, NP). The inter-observer agreement for the scoring of each item for the included studies was determined by the kappa statistics [9]. Based on the power of kappa, the degree of agreement was defined as almost perfect (0.81–1.00), substantial (0.61–0.80), moderate (0.41–0.60), fair (0.21–0.40), or slight (0.00–0.20).

### Data analysis

The baseline data were presented as numbers, proportions, averages, standard deviations (SD), and ranges. We performed the data analysis of the outcomes using Microsoft Excel® (Version 16.55) spreadsheets and STATA (Version 17.0, StataCorp LLC, Texas, USA). Mann–Whitney U-test was used to compare the means. A *p*-value of < 0.05 was considered statistically significant.

## Results

### Study characteristics

Out of 1015 records identified with our search strategy, 1001 were excluded due to irrelevance, non-English language, incomplete data, duplications, or not being original articles (see Fig. 1). 13 studies were included in the analysis [10–22]. All included studies were retrospective in design. The median follow-up time was 3.6 years (IQR 2.2–6.4). The study baseline characteristics and outcome measures are summarised in Table 1.

**Table 1:** The baseline characteristics and outcomes of high-pressure balloon dilatation (HPBD) of primary obstructive megaureters (POM) in the studies included in the current review

Study	n patients	Boys/girls (n)	Laterality of POM left/right/bilateral	Age at first HBPD, mean or median (range)	Length of follow-up, mean or median (range)	Mean AP diameter pre/post operatively (mm)	Mean ureteral diameter pre/post operatively (mm)	Mean T½ (min) pre/post operatively	Mean DRF (%) pre/post operatively	Complications C–D grade 1/2/3/4/5
Destro et al. [16]	30	21/9	20/9/1	Mean 3.6y (0.4–12.2)	Mean 3.3y	NA	16/7.9	NA	NA	1/1/1/0/0
García-Aparicio et al. [18]	20	15/5	12/6/2	Mean 14.18 mo (3–103)	Mean 49 mo (14–80)	24.2/6	15/6.6	NA	NA	NA
Christman et al. [15]	17	10/7	13/4/0	Mean 7.0y (3–12)	Mean 3.2y (2–6.5)	NA	NA	NA	36/NA	NA
Casal Beloy et al. [13]	13	7/6	8/5/0	Mean 9.46 mo, median 9 mo (2–24)	Mean 10.3y, median 10.3y (4.7–12.2)	19.5/8.9	14.7/5.1	80.3/9	50/50	0/4/2/0/0
Torino et al. [22]	12	8/4	9/3/0	Mean 23.5mo, median 14.5mo (5–61)	Median 16.5mo (15–30)	NA	15.9/7.1	NA	NA	0/0/0/0/0
Kassite et al. [19]	33	27/6	16/26/9	Median 14.7mo (3mo–15 yr)	NA	16.7/6	15.3/5.3	NA	NA	0/11/10/0/0
Romero et al. [21]	29	18/11	16/10/3	Median 4.03 mo (1.6–39 mo)	Median 47 mo	18.6/6.6	14.7/8.5	NA/9.2	30/NA	0/5/6/0/0
Angerri et al. [10]	7	6/1	6/1/0	Mean 14.4 mo (5–34)	Mean 31 mo (12–56)	NA	NA	56/30	36/38	0/1/1/0/0
Ortiz et al. [20]	73	58/15	51/22/6	Median 4 mo (0.5–44)	Mean 6.4 y (2.6–10.2)	19.2/10.3	14.9/9.2	NA/9.8	44.4/46.2	0/5/31/0/0
Capozza et al. [12]	12	8/4	8/4/0	Mean 8 mo (6–12mo)	Mean 21mo (2–44mo)	NA	19.4/9.5	NA	NA	0/0/0/0/0
Faraj et al. [17]	42	36/6	30/8/4	Median 12.5 mo (2–128)	Median 35.5mo (12–101)	19.1/13.6	15.7/12.1	NA	54/47	5/3/5/0/0
Chiarenza et al. [14]	38	33/5	24/15/0	Median 23mo (3mo–5.5y)	Median 6.5y (12mo–14y)	NA	NA	NA	NA	NA
Bujons et al. [11]	19	16/3	10/8/1	Mean 17mo (1–44mo)	Mean 6.9y (3.9–13.3 y)	21/4.6	18.1/4.3	69.6/16.5	41.2/41.5	0/0/0/0/0

The means estimated from the medians and ranges were used in the outcomes were appropriate.

*NA*, not available; *DRF*, differential renal function; *AP*, anteroposterior diameter of the renal pelvis; *T½*, washout half time; *C–D*, Clavien–Dindo [23] classification of surgical complications

### Summary of the included studies

#### Angerri et al. [10]

This was a retrospectively conducted study from Spain. Seven children aged from 1 to 3 with the diagnosis of POM were treated with HPBD with a mean follow-up of 31 months. Marked improvement of the obstructive pattern was seen on the renogram in five patients after the primary procedure. One patient presented with a febrile UTI 14 months after the procedure without the presence of VUR. Six of the treated patients (85%) showed an improvement of the obstructive pattern, and secondary dilatation was needed in one case.

#### Bujons et al. [11]

This was a retrospective review from Spain. The study included 19 patients with POM who were treated with HPBD. The mean age of surgery was 17 months, and the mean follow-up of 6.9 years (range 3.9–13.3 years); however, one patient was lost to follow-up. One patient presented with UTI and Grade II VUR 14 months after the endoscopic dilatation, but at follow-up, the patient presented without symptoms or a loss of renal function. Significant improvement was shown after the procedure in the average time of elimination on the MAG-3 renogram (*p* < 0.001). After the first dilatation, the overall success rate was 90%. One patient proceeded to second HPBD due to persistent hydroureteronephrosis, and the procedure was successful.

#### Capozza et al. [12]

Conducted in Italy, this was a retrospective study. Twelve patients with POM, mean age of 8 months (range 6–12 months), were treated with balloon dilatation. The mean follow-up was 21 months. Ten of the patients presented with a clear stenotic ring, and HPBD was performed in seven patients. In the three cases with a persistent ring, a cutting balloon ureterotomy (CBU) was performed, resulting in the immediate and complete disappearance of the stenosis. Two patients presented without a stenotic ring in the procedure and showed no improvement after the procedure. The overall success rate of patients treated endoscopically with HPBD and CBU was 83%. No postoperative complications were observed.

#### Casal Beloy et al. [13]

This was a retrospective review from Spain. The review included thirteen patients diagnosed with POM treated with HPBD and double-J stenting. The patients' ages ranged from 2 to 24 months (mean 9 months). The mean follow-up was 10.3 years. After the procedure, one double-J stent was replaced due to malpositioning, and in one case, removal of the double-J stent was needed due to UTI 3 weeks after the procedure. A total of four patients presented with UTI postoperatively. Postoperative MAG-3 diuretic renogram showed no signs of obstruction in all the patients. Comparison of pre-and postoperative T½ on MAG-3 diuretic renogram revealed statistical differences (*p* < 0.001).

#### Chiarenza et al. [14]

This study was published from Italy and was retrospective. Thirty-eight patients with POM from 2005 to 2018 were included. The mean follow-up was 6.5 years, and the mean age of 23 months (range 3 months–5.5 years). HPBD was successfully performed in 23 patients. In nine cases, failure to pass the guidewire through vesicoureteral junction prevented the endoscopic treatment, and further treatment with open ureteral reimplantation was needed. No stenotic ostium was identified during the procedure, and HPBD was not performed in six patients. The patients HPBD was performed in were divided into groups according to the length of the stenotic tract. Four patients with stenotic tract under 5 mm presented resolution after the first treatment, while in one case, secondary dilatation was needed. Patients with a stenotic tract from 5 to 10 mm were found to be in an intermediate prognosis group; four patients were successfully treated with the first HPBD, while seven patients presented resolution after the second HPBD, and one patient after the third endoscopic treatment. All patients with a stenotic tract over 10 mm underwent ureteral reimplantation during follow-up.

#### Christman et al. [15]

This was a retrospective review from the USA. A total of 17 patients with POM were treated with HPBD. The mean age of the patients was 7.0 years (range 3–12 years), and the mean follow-up was 3.2 years (range 2.0–6.5 years). For patients with a narrowed distal ureteral segment shorter than 2 cm, balloon dilation was performed, and if the segment was 2–3 cm, laser incision was done. Marked improvement of hydroureteronephrosis was observed in 12 patients (71%). The five remaining patients had some improvement, and MRU revealed no evidence of obstruction. All patients remained symptom-free during the follow-up time. Recurrent urolithiasis was observed in two patients.

#### Destro et al. [16]

Conducted in Italy, this was a retrospective study. A total of 30 patients were treated with HPBD with double-J stent placement, mean age of 3.6 years (range 0.4–12.2 years). There were difficulties in inserting the guidewire in three patients. The mean follow-up was 3.3 years, and during follow-up, 5 patients underwent ureteral reimplantation. Transient hematuria and UTI were diagnosed in 1/30 patients (3%), and none of the patients developed VUR. Statistical differences were found in pre-operative and postoperative ureteral diameters (*p* = 0.0009).

#### Faraj et al. [17]

This was a retrospective comparison study from France. A total of 42 patients with 46 affected ureters with POM were treated with HPBD. The patients were divided into two groups: double-J stent was placed on 16 patients, and 26 underwent endoscopic dilatation without double-J stent placement. Statistical differences were found regarding the postoperative complications (Clavien–Dindo III): 31% in the group with double-J stent placement and 0% in the group with no double-J stent placement. The overall success rate after the first dilatation was 79%, and no statistical difference was found between the two groups. Nine patients progressed to ureteral reimplantation, one had VUR after the procedure, and one progressed to nephrectomy due to recurrent febrile UTIs 15 months after the procedure. In both groups, there was a significant improvement in ureteral diameter before and after the surgery (*p* = 0.0084). The follow-up was 70 months for the group with double-J stents and 26 months for those without a double-J stent.

#### García-Aparicio et al. [18]

This was a retrospective review from Spain. Twenty patients with a total of 22 ureters with POM were treated with HPBD, the mean age at surgery being 14.18 months (range 3–103 months), and the mean follow-up was 49 months (14–80). 86.4% of the ureters treated showed improvement of hydroureteronephrosis. Six ureters developed VUR postoperatively, and four ureters progressed to ureteral reimplantation. Dilation was repeated in five patients due to the inability to pass the cystoscope through the vesicoureteral junction as the double-J catheter was removed. At six months, all patients were free from obstruction or VUR in the US scan. The presence of paraureteral diverticula in the preoperative VCUG and patients with bilateral POM increased the risk of postoperative VUR (*p* < 0.05).

#### Kassite et al. [19]

This was a retrospective study conducted in France that involved 33 patients with POM. A total of 42 ureters were treated with HPBD and double-J stenting, with a mean age of 14.7 months (range 3 months–15 years). The mean follow-up was 24 months (2 months–5 years). After the primary endoscopic treatment, 86% of the ureters showed improvement of hydroureteronephrosis. Secondary endoscopic treatment was needed in three patients, and four patients progressed to surgical treatment due to worsening of the hydroureteronephrosis. 13 patients (61%) had a febrile UTI after HPBD during the stenting period, and 11 of the infections were related to double-J stenting. Ureteral stent migration occurred in two patients. Statistical analysis showed significant improvement in the diameter of the ureter and pelvis after surgery.

#### Ortiz et al. [20]

This was a retrospective study from Spain. A total of 73 patients and 79 ureters affected by POM were included. All the patients underwent HPBD with double-J stent placement. The mean age at surgery was four months (range 15 days–3.6 years), with a mean follow-up was 6.4 years. MAG-3 renogram showed a significant improvement in renal drainage after HPBD (*p* < 0.001), and significant improvement was observed in hydronephrosis and ureteral diameter after the treatment (*p* < 0.001). Seventeen patients (21.5%) developed secondary VUR, and nine needed a second endoscopic treatment due to re-stenosis. In 10 cases (12.7%), secondary ureteral reimplantation was needed.

#### Romero et al. [21]

This was a retrospective review from Spain. Twenty-nine children with 32 renal units diagnosed with POM were treated with semi-compliant high-pressure balloon dilatation and double-J stenting. The mean age at the procedure was 4.04 months (range 1.6–39 months), with a mean follow-up of 47 months. In three cases, the endoscopic procedure was not successful; in two cases, due to difficulties in passing the guidewire through the VUJ; and in one case, due to stent migration. These cases underwent ureteral reimplantation. Twenty patients with 23 renal units (69%) showed improvement of hydroureteronephrosis and drainage on ultrasonography and MAG-3-Lasix 18 months after the procedure. Two patients progressed to secondary HPBD due to persistent hydroureteronephrosis, and two other patients were treated with open ureteral reimplantation. Five patients were diagnosed with UTI and secondary VUR during follow-up, which in two cases led to ureteral reimplantation.

#### Torino et al. [22]

This was a retrospective review conducted in Italy, including 14 patients with POM. Twelve patients (86%) were treated with HPBD and double-J stenting, and two patients were treated with open ureteral reimplantation due to the failure to pass the balloon catheter through the VUJ. The mean age of the patients was 14.5 months (range 5–61 months), with a mean follow-up of 16.5 months (range 15–30 months). All patients improved postoperatively on the ultrasonographic scan, and no evidence of obstruction or VUR was detected on postoperative MAG-3 renography. A significant decrease in ureteral dilatation was seen at the postoperative ultrasound on all patients (*p* = 0.0005). No operative complications were observed during follow-up period.

### Technical aspect of high-pressure balloon dilatation

The authors reported using rigid cystoscopes varying in size from 8 to 10.5 Fr. The maximum diameter of the dilated balloon used varied from 2 to 10 mm, with the most common diameter being 4–6 mm. The length of the balloon was either 2 or 4 cm. The time the balloon was kept dilated varied greatly (from 2 to 15 min), as did the pressure (8–20 ATM).

Out of 13 studies included, ten reported routinely using a double-J stent for three weeks to 3 months after HPBD [10, 11, 13, 15, 16, 18–22]. Two authors [12, 14] reported that a double-J was not inserted routinely. Faraj S et al. [17] compared those with and without double-J after HPBD. They reported similar success rates in those with double-J stent (75%) and those without (81%). However, significantly more complications were reported in those with double-J stent (56% and 15% for all Clavien–Dindo grades, respectively, *p* = 0.014).

Out of the 13 studies, five [10, 14, 19–21] reported using a single perioperative antibiotics prophylaxis only, while five [13, 15, 17, 18, 22] reported continuing antibiotics as chemoprophylaxis after HPBD. Most used prophylaxis until the double- J stent was removed, while Casal Beloy et al. [13] reported continuing antibiotics for only 5 days. No data on antibiotic prophylaxis was given in three studies [11, 12, 16].

### Quality assessment

The assessment of methodological quality of the studies is depicted in detail in Table 2. The average scores of the included studies ranged from 11 to 16. The study by Romero et al. [21] had the minimum risk of bias and the study by Chiarenza et al. [14] had the maximum risk of bias. The risk was high in eight studies [10, 12–16, 18, 22] and moderate in the remaining five studies [11, 17, 19–21]. The inter-observer agreement for the quality assessment was good (kappa: 0.88).

**Table 2:** The methodological quality of the included studies (n = 13) on high-pressure balloon dilatation of primary obstructive megaureters

Study	Reporting	External validity	Internal validity bias	Internal validity confounding	Power	Total scores	Mean
Destro et al. [16]	8/7	3/3	4/4	1/0	0/0	16/14	15
García-Aparicio et al. [18]	8/6	3/3	4/3	1/0	0/0	16/12	14
Christman et al. [15]	7/6	3/3	3/3	1/0	0/0	14/12	13
Casal Beloy et al. [13]	8/8	3/3	3/2	0/0	0/0	14/13	13.5
Torino et al. [22]	9/7	3/3	4/2	1/0	0/0	17/12	14.5
Kassite et al. [19]	8/8	3/3	4/4	1/0	0/0	16/15	15.5
Romero et al. [21]	9/9	3/3	4/3	1/0	0/0	17/15	16
Angerri et al. [10]	8/5	3/3	3/2	1/0	0/0	15/10	12.5
Ortiz et al. [20]	8/8	3/3	4/3	1/1	0/0	16/15	15.5
Capozza et al. [12]	9/6	3/3	4/3	1/0	0/0	17/12	14.5
Faraj et al. [17]	9/9	3/3	4/3	1/0	0/0	17/15	16
Chiarenza et al. [14]	8/3	3/3	3/1	1/0	0/0	15/7	11
Bujons et al. [11]	9/8	3/3	4/3	1/0	0/0	17/14	15.5

The kappa value for the inter-observer agreement of the quality assessment was 0.8755

### Primary outcome measures

Balloon dilation seems effective in reducing hydroureteronephrosis. Derived from the ten studies [11–13, 16–22] with data on ureteral diameters (POM n = 292), the mean ureteral diameter reduced from preoperative 15.8 mm (range 2–30) to 8.0 mm (0–30) at postoperative follow-up (*p* = 0.00009). According to 7 studies [11, 13, 17–21] reporting the mean anteroposterior pelvic diameter (AP) pre- and postoperatively (POM n = 245), AP diameter reduced from 16.7 mm (0–46) to 9.7 mm (0–36) (*p* = 0.00107).

Only one study [20] reported parenchymal thickness both pre- and postoperatively (n = 49); parenchymal thickness was 4.1 mm (± 1.6) preoperatively, 5.5 mm (± 2.2) 3 months after balloon dilatation, and 8.3 mm (± 2.4) at the last follow up (1.5–13.5 years) which was reported to be a statistically significant increase (*p* < 0.001 in T-test).

None of the included studies systematically described hydronephrosis according to the Society of Fetal Urology classification, either pre- or postoperatively, except for the study by Garcia-Aparicio et al. [18].

Differential renal function (DRF) was reported preoperatively in seven studies [10, 11, 13, 15, 17, 20, 21] (POM n = 200) and postoperative in five studies [10, 11, 13, 17, 20] (POM n = 153). The five studies reporting DRF both pre- and postoperatively showed similar DRF after balloon dilation, 47.0% (range 22–57) to 46.1% (range 10–58), *p* = 0.9765.

There was little data available on the effect of balloon dilatation in relieving obstruction. The mean preoperative washout halftime (T½) was 70.7 min (range 25–150 min) and 16.4 min (4–86 min) postoperatively according to 3 studies [10, 11, 13] with n = 39 POM. The statistical significance could not be determined due to the small number of studies reporting T½ pre- and postoperatively.

The success rate after one HPBD was 71% (251/353 patients), 79% (279/353) after two HPBDs, and 79% (280/353) after three HPBDs, meaning no other interventions such as open surgery, endoscopic treatment of VUR, or treatment of POM with cutting balloon were required for these patients. Many authors report that they only attempted HBPD once or twice, and then decided on open surgery. Open ureteral reimplantation surgery was performed for 16.4% (58/353) of the patients.

### The secondary outcome measures

Our secondary outcome was to study the complication rate of endoscopic high-pressure balloon dilatation. A complication rate of 33% (n = 92/277) after balloon dilatation was calculated from 10 studies [10–13, 16, 17, 19–22] when all grade I-V complications according to the Clavien–Dindo classification were included [23]. While no life-threatening (grade IV–V) complications were reported, 20% (56/277 patients) were reported needing intervention under general anaesthesia (i.e., grade 3b complication) after balloon dilatation. Milder, grade II and I complications were reported in 30 and 6 patients.

The most typical complication after balloon dilatation was postoperative urinary tract infection (UTI). The rate of postoperative UTI was reported to be 12% (33/274) according to ten of the studies [10, 12, 13, 15–17, 19–22].

Most authors did not report routinely screening for vesicoureteral reflux (VUR) after balloon dilatation. The commonest protocol seemed to be to screen those patients with postoperative UTIs after the stent removal. The rate of postoperative VUR was reported in 25/319 (7.8%) patients in 11 studies [10–12, 14–17, 19–22].

### HPBD for children under 1 year of age

Subgroup analysis of patients aged less than 12 months at the time of first HPBD showed similar results to the ones reported above. HPBD seems to reduce both hydroureteronephrosis and T½, and DRF seems to be maintained. However, due to the small numbers of infants (n = 31), the statistical significance of these findings could not be confirmed.

In children less than one year of age, the mean anteroposterior diameter of the renal pelvis was 20.1 mm preoperatively and 9 mm after HPBP, according to one study (n = 11) [13]. The mean ureteral diameter was 16.3 mm preoperatively and 7.0 mm postoperatively in three studies [12, 13, 22] with 25 infants.

The differential renal function was 44.9% preoperatively and 46.6% postoperatively, according to four studies [10–13] with 31 infants. T½ in infants was 70 min and 13 min in 3 studies [10, 11, 13] with 22 infants, respectively.

## Discussion

We show in this systematic review that endoscopic high-pressure balloon dilatation can be safely utilised for treating symptomatic primary obstructive megaureter. With this mini-invasive technique, one can potentially avoid open surgery that may cause permanent damage to bladder function and be technically demanding, especially in young patients. HPBD significantly decreased both ureteral diameter and the anteroposterior diameter of the renal pelvis. We observed a complication rate of 33%, but no Clavien–Dindo grade IV–V complications were reported. The median follow-up time was 3.6 years. Postoperative infections and VUR were detected in 12% and 7.8% of cases, respectively. For children under 1 year of age, HPBD seems safe with similar outcomes to older children.

Primary megaureter is the second most common cause of hydronephrosis in newborns, with an incidence of 0.36 per 1000 live births [24]. It is more common in boys than in girls and on the left side [25]. The rate of bilateral involvement is approximately 30 percent [26]. The pathogenesis of primary megaureter appears to be most commonly due to an abnormality or delay in the muscular development of the distal ureter resulting in the formation of an aperistaltic segment and functional obstruction [27]. It can be divided into four types according to the presence or absence of reflux and obstruction. The division between obstructive and non-obstructive megaureter is not always clear and currently, obstruction is defined as a restriction of urinary flow that will affect renal homeostasis [28]. Most cases of prenatally diagnosed POM resolve spontaneously. Surgical correction is only required in 15–20% of cases due to symptoms (recurrent urinary tract infection, pyelonephritis, persistent flank pain, calculi, or haematuria) or renal function deterioration during the follow-up [29]. Traditionally, surgery has entailed ureteral reimplantation, but HPBD has raised interest, especially in patients under one year of age. Reports on the complication risks of open ureteral reimplantation for patients under one year of age vary [30].

Regarding open surgery for primary megaureter, DeFoor et al. reported a success rate of 82% in a cohort of children with a mean age of 4.9 years [4]. Peters et al. [31] presented a series of 47 infants less than eight months of age, of which 42 underwent open surgery for POM. Of these, 8 had postoperative VUR, of which three underwent repeat reimplantation, and five patients had mild obstruction postoperatively, of which two required repeat reimplantation. The morbidity associated with open reimplantation surgery for POM has been reported to be 4–25%, with the most significant harms presenting as ureteral stricture, VUR, and transient postoperative voiding dysfunction [32–36]. Reports on laparoscopic and robotic correction of POM in children are still relatively rare. Small series have been published treating POM with laparoscopic [37] and robotic ureteral reimplantation [38–40], and results seem comparable to those of open repair. However, more data and long-term follow up studies are needed to ascertain this.

Since Angulo et al. first reported HPBD in treating POM in 1998, it has become the primary treatment method in many centres [5]. HPBD is tempting as the bladder wall is not compromised and will not violate ureteral circulation. Further, endoscopic treatment does not impact possible future bladder surgery. Despite long-term use of HPBD, high-quality supporting evidence is scarce. Randomised comparative trials are lacking, and many clinical studies are limited in study design. The risk of bias of the studies analysed here was moderate or high, and the quality of reporting was moderate. The success rate of HPBD was estimated to be 71–79%, with a complication rate of 33%. Most complications were mild (Clavien–Dindo grades I–II), but reintervention in anaesthesia was required in 20% of cases. No life-threatening complications were reported.

While the effect on both ureteral diameter and renal pelvic AP diameter were commonly reported, the treatment effect on obstruction and renal parenchymal thickness were seldom addressed. Only Ortiz et al. [20] reported parenchymal thickness both pre- and postoperatively. However, DRF remained stable after HPBD according to the 5 studies reporting DRF both pre- and postoperatively [10, 11, 13, 17, 20].

Doudt et al. found a success rate of 70% for HBPD in the treatment of POM. The success rate was 60–75% for infants, for whom endoscopic treatment was sometimes considered temporising. Identifying those patients, who only need temporising procedures (e.g., double-J stents, ureterocutaneostomy, nephrostomy) from those infants who will need definite intervention is challenging. The complication rate of HPBD is between 12.0 and 28.7%, as deduced from previous systematic reviews with mean follow-up times of 3.7 and 3.2 years, respectively [6, 41]. Most complications have been classified as mild (Clavien–Dindo grades I–II). The rate of surgical reintervention after HPBD was estimated to be 36.7%, and 17.3% needed ureteral reimplantation [6].

Currently, it seems customary to use double-J stents after HBPD. Most of the studies analysed here [10, 11, 13, 15, 16, 18–22] reported routinely placing a double-J stent after balloon dilatation and keeping it in for three weeks to 3 months. Chiarenza et al. and Capozza et al. [12, 14] reported that no double-J stents were placed after high pressure balloon dilatation. Interestingly, Faraj S et al. [17] compared those with and without double-J stents after high-pressure balloon dilatations reports and showed similar success rates of balloon dilatation in those with double-J stent (75%) and those without (81%) but more complications in those with double-J stent. They report an overall complication rate of 56% and 15% in those without double-J stents (*p* = 0.014). The rates of Clavien–Dindo grade III complications only in the same study were 31% and 0%, respectively (*p* = 0.0051).

This study is limited by several factors. First, there is a variable reporting of the outcomes in the included studies and overall reporting in them was incomplete. Second, most of these studies were retrospective observational studies and had a moderate or high risk of bias. Third, the studies presented only a limited number of patients. Fourth, all studies lacked comparative control groups.

## Conclusion

Although a few systematic reviews have been published previously on HPBD, it is noteworthy that numerical data on treatment effects has been incomplete. The current review presents a limited quantitative analysis of treatment effects and complications of HPBD. Due to limitations in the quality of the included studies, a proper meta-analysis was deemed unreliable. Nevertheless, the results from this study indicate that HPBD appears to be safe and can be used as a first-line treatment for symptomatic POM. Because there are little data available, further comparative studies are needed addressing the treatment effect in infants and the treatment's long-term outcomes. Due to the nature of POM, identifying those patients who will benefit from HPBD remains challenging.

## Data Availability

The datasets used and/or analysed during the current study are available from the corresponding author on reasonable request.
